# THE WHATMATTERS APP: CO-DESIGNING PERSON-CENTERED CARE FOR PEOPLE WITH DEMENTIA IN CARE SETTINGS

**DOI:** 10.1093/geroni/igac059.2784

**Published:** 2022-12-20

**Authors:** Lillian Hung, Ellen Guo, Mariko Sakamoto, Karen Lok, Yi Wong, Candy Tran, Jim Mann, Annette Berndt, Eva Egeberg

**Affiliations:** University of British Columbia, Vancouver, British Columbia, Canada; The University of British Columbia, University Endowment Lands, British Columbia, Canada; University of British Columbia, Vancouver, British Columbia, Canada; University of British Columbia, Vancouver, British Columbia, Canada; University of British Columbia, Vancouver, British Columbia, Canada; School of Nursing University of British Columbia, University Endowment Lands, British Columbia, Canada; Community Engagement Advisory Network, Vancouver, British Columbia, Canada; Community Engagement Advisory Network, Vancouver, British Columbia, Canada; Emily Carr University, Vancouver, British Columbia, Canada

## Abstract

The purpose of our study is to provide comfort through digital resources (e.g., music and visual materials) for patients/residents with dementia in hospitals and long-term care. By working with users (patients/residents, families, and staff) and using a co-design approach, we have developed a mobile app prototype called “WhatMatters” to equip staff with a useful digital tool for delivering person-centered care in hospitals and long-term care homes. Using user experience co-design methods, we conducted a series of virtual co-design workshops with acute and long-term care staff (n=10), clinical experts (n=3), residents (n=3), and patient and family partners (n=7) to understand: (a) what “comfort” means, (b) how care needs are communicated and provided for, and (c) how a mobile app may be used to support psychosocial needs of people living with dementia in hospital and long-term care settings. Thematic analysis has identified three themes to inform the development of the mobile app WhatMatters: (a) familiarity brings comfort, (b) sharing of information between staff and families allows for continuity of care and person-centered care, and (c) accessible and curated content can evoke memories and create a comforting space. Our study conclusions are: It is feasible and necessary to work with users (including clinical staff, patient, and family partners) and relevant stakeholders to co-design a mobile app, a useful tool to support the delivery of person-centered care in care settings.

